# Smart Red Blood Cell Carriers: A Nanotechnological Approach to Cancer Drug Delivery

**DOI:** 10.3390/cimb47090711

**Published:** 2025-09-01

**Authors:** Ioannis Tsamesidis, Georgios Dryllis, Sotirios P. Fortis, Andreas Sphicas, Vasiliki Konstantinidou, Maria Chatzidimitriou, Stella Mitka, Maria Trapali, Petros Skepastianos, Anastasios G. Kriebardis, Ilias Pessach

**Affiliations:** 1 Department of Biomedical Sciences, Faculty of Health Sciences, International Hellenic University, Sindos, 57400 Thessaloniki, Greece; themikonstantinidou@yahoo.gr (V.K.); mchatzid952@gmail.com (M.C.); mitkast@hotmail.com (S.M.); pskep@otenet.gr (P.S.); iliaspessach1980@gmail.com (I.P.); 2 Laboratory of Reliability and Quality Control in Laboratory Hematology (HemQcR), Department of Biomedical Sciences, School of Health & Caring Sciences, University of West Attica (UniWA), 12243 Egaleo, Greece; sfortis@uniwa.gr (S.P.F.); andreas.sphicas@gmail.com (A.S.); ymaria@uniwa.gr (M.T.); akrieb@uniwa.gr (A.G.K.)

**Keywords:** erythrocytes, nanoparticles, drug delivery systems, cancer therapy

## Abstract

The efficient and targeted delivery of pharmaceutical substances remains a major challenge in modern therapeutics. Traditional drug delivery systems often suffer from limited bioavailability, rapid clearance, and off-target effects. Red blood cells (erythrocytes), due to their long circulation time, biocompatibility, and immune-evasive properties, have emerged as promising carriers in the development of novel nanotechnology-based drug delivery platforms.A comprehensive literature review was conducted, analyzing recent studies on erythrocyte membrane-coated nanoparticles, their interactions with loaded therapeutic agents, and their performance in vitro and in vivo. Special focus was given to applications in chemotherapy, photodynamic therapy (PDT), photothermal therapy (PTT), and immunotherapy. Erythrocyte-based nanocarriers demonstrated improved circulation times, reduced immune clearance, and enhanced targeting capabilities compared to traditional nanoparticles. Encapsulation of nanoparticles within erythrocyte membranes preserved the functional integrity of the carrier while minimizing systemic toxicity. However, challenges such as membrane stability, hemocompatibility, and the potential for nanoparticle-induced hemoglobin dysfunction were identified as areas requiring further research. In conclusion, erythrocyte membrane-coated nanoparticles represent a unique and promising strategy for drug delivery, combining the natural advantages of red blood cells with the versatility of nanotechnology.

## 1. Introduction

Cancer remains one of the leading causes of death worldwide, affecting millions of lives across all nations regardless of economic status, age, or gender [1]. It encompasses a wide range of diseases characterized by the uncontrolled growth and spread of abnormal cells, which can invade nearby tissues and organs [2]. The most common types are carcinomas, which begin in the skin or tissues that line internal organs, including breast, lung, prostate, and colon cancers [3]. Sarcomas are cancers that arise from connective tissues, including bone, muscle, and fat [4]. Leukemias are cancers of the blood and bone marrow, marked by the rapid production of abnormal white blood cells [5]. Lymphomas affect the lymphatic system, while myelomas originate in plasma cells of the immune system [6]. Cancers of the central nervous system, including brain and spinal cord tumors, present unique challenges due to their location [7]. Other less common types include germ cell tumors, which typically develop in the reproductive organs, and neuroendocrine tumors, which arise from hormone-producing cells [8]. Despite their differences, all types of cancer share the hallmark characteristic of abnormal, uncontrolled cell growth [3]. Despite advancements in medical research and treatment, many forms of cancer continue to pose significant challenges to health systems due to late diagnoses and limited access to care in some regions [9]. The pathophysiology of cancer involves a complex series of genetic and cellular events that disrupt normal cell regulation and lead to malignant transformation [10]. Cancer fundamentally arises when genetic mutations develop in a cell’s DNA, disrupting the normal regulation of cell growth, division, and death. These mutations often involve oncogenes, tumor suppressor genes, or DNA repair genes. They may result from environmental exposures (such as tobacco smoke or radiation), infections, inherited genetic alterations, or random errors during cellular processes [11]. As these mutated cells evade the body’s normal regulatory mechanisms, they begin to proliferate uncontrollably, ignoring signals that usually tell cells to stop dividing or to undergo apoptosis (programmed cell death). Over time, they accumulate additional mutations that further enhance their survival and invasive capabilities. These cancerous cells can form a mass of tissue, known as a tumor, and in malignant cancers, they can invade surrounding tissues and spread to distant parts of the body through the blood and lymphatic systems, a process known as metastasis. This ability to metastasize is a hallmark of cancer and one of the main reasons it becomes life-threatening [12].

Modern medicine has developed numerous methods of drug delivery for treating diseases of all kinds, including cancer [13]. Starting with conventional methods of exogenous drug administration, such as intravenous, intramuscular, subcutaneous infusion, and oral administration of active substances, these seem to act effectively, providing beneficial properties to subjects receiving the drug [14]. However, not all conditions and diseases are easily managed by their nature, a typical example being malignant solid tumors in vital organs. Whenever a tumor is surgically removed, complementary treatment such as radiotherapy, chemotherapy, immunotherapy, or hormone therapy is almost always required. This additional treatment may serve either a therapeutic or palliative purpose, ultimately aiming to improve life expectancy and quality of life [15]. Although recent decades have brought remarkable progress in the administration of pharmaceutical agents, there are still many cases where conventional treatments are ineffective, or their results are insufficient to provide relief or achieve complete cure in later stages [16]. In recent years, a lot of research has been exercised in finding ways and methodologies, with the aim of the effective distribution and release of drugs against malignant tumors and cancer cells. Recent research indicates that the application of nanomaterials, nanotechnology, and nanomedicine in cancer treatment holds great potential due to their ability to enhance drug targeting. Drug delivery techniques based on nanoparticles have proven exceptionally successful in releasing different medications at different sites. We review in this work, some of the most important delivery methods that have been developed, a schematic approach below illustrates the complex connection between nanoparticles and cell membranes in targeting cancer [17].

In more details, a lot of attempts to use nanoparticles as pharmacological carriers in chemotherapy was performed, considering that the most common therapeutic strategies in oncology present several limitations [18]. These include significant side effects and the non-selective destruction of healthy immune cells, often without effectively targeting malignant cells. To address this, early efforts focused on encapsulating and “packaging” drugs within nanoparticles [19]. This approach aimed to increase drug stability and prolong its viability, allowing for gradual release of the active substance while minimizing adverse effects on patients. The primary strategy to enhance the pharmacokinetic profile of nanoparticles involved modifying their membranes by incorporating hydrophilic polymers such as polyethylene glycol (PEG) and poly-2-oxazolines [20]. These polymers are well known for their biocompatibility and low toxicity. However, despite these modifications, nanoparticles still exhibit limited specificity and binding to target tissues—even when engineered with surface-bound targeting proteins. This shortcoming is largely due to their rapid clearance from the bloodstream by the liver and spleen [21]. As a result, researchers turned their attention to blood cells, which naturally circulate in the bloodstream without being prematurely removed. This led to the development of a new strategy focused on designing a “transport vehicle”, a biological carrier capable of shielding nanoparticles from degradation and extending their circulation time in the bloodstream.

This review explores the emerging field of using erythrocytes (red blood cells) as carriers for nanoparticle-based drug delivery systems, particularly in the context of anticancer therapies. It highlights the advantages of loading nanoparticles into erythrocytes, aiming to improve the pharmacokinetic properties and reduce the immunogenicity of therapeutic agents. Special attention is given to the effects of nanoparticles on red blood cell hemocompatibility, their interactions with erythrocyte membranes, and the subsequent impact on membrane proteins and cell morphology. Further sections delve into how nanoparticle integration may alter key physiological functions of erythrocytes, such as oxygen transport and antioxidant defense mechanisms. The review also examines innovative strategies, including the development of biomimetic and camouflaged nanoparticles using red cell membranes, to enhance circulation time and targeting efficiency. Finally, it discusses the potential of these erythrocyte-based systems to advance anticancer therapies by improving drug delivery specificity and minimizing systemic side effects.

## 2. Methods

A comprehensive literature search was conducted across the databases PubMed, Scopus, and Google Scholar. The primary keywords guiding the search were “nanoparticles” and “erythrocytes.” The search strategy was subsequently refined to focus on studies investigating nanoparticles loading into red blood cells, the interactions between nanoparticles and erythrocyte membranes, and the development of biomimetic nanoparticles utilizing erythrocyte components. To capture a broader range of relevant studies, additional search combinations were employed, including “nanoparticles AND erythrocytes,” “nanoparticle encapsulation AND red blood cells,” “red blood cell membrane AND drug delivery,” “erythrocyte-based nanoparticles AND anticancer therapy,” and “biomimetic nanoparticles AND circulation time.” Only articles published in English were considered. The period for data collection and interpretation spanned from June 2024 to March 2025.

## 3. Results

### 3.1. Nanotechnology and Nanoparticles

Nanotechnology is the science, engineering, and technology conducted at the nanoscale typically between 1 and 100 nanometers where unique physical and chemical phenomena enable novel applications across diverse fields such as chemistry, physics, biology, medicine, engineering, and electronics [22]. One of the major challenges in this domain involves the production and analysis of extremely small structures, sometimes comprising only tens to thousands of atoms. Given its vast potential, nanotechnology has become a major area of investment globally, particularly in technologically advanced regions such as China, the United States, and Europe [23]. Nanoscience and nanotechnology encompass the study and application of systems, structures, and technologies that exhibit distinctive capabilities due to their nanoscale dimensions. The term “nano” originates from the Greek word meaning “dwarf” or “extremely small,” and in scientific terms, it denotes one-billionth of a meter (10^−9^ m) [24]. Nanoparticles (NPs) are materials with at least one dimension smaller than 100 nanometers. These particles, whether natural or synthetic, are typically spherical in shape and are commonly composed of polymers [25]. Their small size and high surface area to volume ratio give them a wide range of potential applications. NPs can improve the solubility and stability of encapsulated substances, enhance transport across biological membranes, and prolong circulation times in the body, thereby improving safety and efficacy [26]. Nanoparticles can be uniform in structure or composed of multiple layers. In multilayered particles, the structure generally includes the following: a surface layer made up of small molecules, metal ions, surfactants, or polymers; a shell layer composed of a material chemically distinct from the core; and a core layer, which forms the central part of the nanoparticle [27]. These particles possess several key physical properties: high mobility in their natural form, a large specific surface area, and the potential to exhibit quantum effects. These properties are dependent on the shape of the particles and find uses in fields such as data storage, optics, and catalysis [28]. The size and shape of nanoparticles are critical to their unique behaviors, affecting their optical, electrical, and chemical properties, as well as their stability, melting point, and density. Because of these characteristics, nanoparticles are increasingly used in a wide range of sectors including biomedicine, electronics, optics, and agriculture. Based on their composition, nanoparticles are generally categorized into three main types: inorganic, carbon-based, and organic. In recent years, nanoparticle-based therapies have gained significant importance in the medical field, with more than 30 formulations based to nanotechnology already approved and over 100 clinical trials currently in progress [29,30].

### 3.2. Advantages of Loading Erythrocytes with Nanoparticles

**Figure 1:**
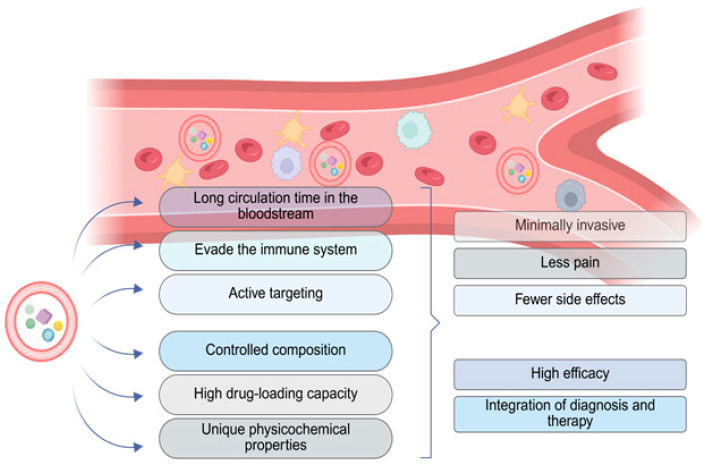
Benefits from the use of nanoparticles that have been loaded with therapeutic agents and incorporated into RBCs, compared to conventional drug delivery methods. Created in BioRender. Fortis, S. (2025) https://BioRender.com/0645avy.

Zhang et al. focused on the encapsulation of overloaded nanoparticles within erythrocytes through their cell membranes. This approach appears highly promising, as erythrocytes offer several critical advantages over individual nanoparticles due to their unique physicochemical properties [31]. They have active targeting, high-drug loading capacity, and controlled composition. Furthermore, they are minimally invasive, cause less pain, and are capable of integrating either diagnosis or therapy (Figure 1). Though, the most fundamental benefit is their ability to evade the immune system, preventing immune attacks and enabling prolonged circulation in the bloodstream, further enhanced by the long lifespan of red blood cells (approximately 120 days). Additionally, erythrocytes demonstrate high biocompatibility and bioavailability, minimizing the risk of toxic reactions often associated with the introduction of exogenous agents (Figure 1). Their abundance, combined with a favorable membrane to cytoplasmic volume ratio, allows for a substantial drug payload to be loaded into each cell. This characteristic not only stabilizes the nanoparticles but also extends their intracellular residence time and reduces the likelihood of nanoparticle aggregation [32]. Moreover, specific features of erythrocytes, including their structural and surface proteins, have been exploited to enhance the design of next-generation drug delivery platforms. These platforms aim to leverage the inherent benefits of erythrocytes, particularly the unique properties of their plasma membranes. In a pilot study, nanoparticles encapsulated within red blood cell (RBC) membranes remained in circulation for approximately 39.6 h, compared to only 15.8 h for nanoparticles coated with polyethylene glycol (PEG) membranes [33]. These findings highlight the significant advantages of erythrocyte membrane coating, notably in extending circulation time and reducing clearance by the reticuloendothelial system [34].

### 3.3. Hemocompatibility of Nanoparticles

Hemocompatibility refers to the compatibility of a material or device with blood and its components, encompassing the ability of the material to perform its intended function without inducing adverse effects such as thrombosis, hemolysis, inflammation, or immune reactions [35]. It is a critical parameter for evaluating the safety and efficacy of biomaterials that come into direct or indirect contact with blood, including vascular grafts, stents, heart valves, dialysis membranes, and drug delivery systems [36,37]. The assessment of hemocompatibility involves a comprehensive analysis of interactions between the material surface and blood elements, including erythrocytes, leukocytes, platelets, plasma proteins, and the coagulation cascade [35]. Key indicators include platelet adhesion and activation, complement system activation, hemolysis rates, coagulation time, and cytokine release. These interactions are influenced by the physicochemical properties of the material, such as surface roughness, hydrophilicity/hydrophobicity, surface charge, and chemical composition [38]. Achieving optimal hemocompatibility requires careful material design and surface engineering to minimize unfavorable blood responses while maintaining functionality [38]. This is especially crucial for long-term implants or devices with prolonged blood contact, where poor hemocompatibility can lead to serious complications such as thrombogenesis, embolism, or chronic inflammation [39].

Once the hemocompatibility of red blood cells (RBCs) is ensured, the subsequent step involves the incorporation of nanoparticles into or within the RBC membranes. This strategy aims to harness the biocompatibility, long circulation time, and immune-evasive properties of RBCs to enhance the therapeutic efficacy and systemic stability of the nanoparticle-based delivery systems [40,41].

Numerous studies have focused on identifying the functionality of nanoparticle use when they are encapsulated within or attached to RBC membranes [35,42,43,44]. To effectively harness erythrocytes as carriers for drug-loaded nanoparticles, a comprehensive understanding of their morphological, biochemical, and biomechanical properties is essential [45]. These include membrane elasticity, surface charge, antigenic profile, and their ability to maintain structural integrity under physiological shear stress. Such knowledge is crucial not only for preserving erythrocyte function during and after nanoparticle loading, but also for optimizing circulation time and targeting efficiency. This underscores the critical importance of establishing and thoroughly characterizing the hemocompatibility between administered nanoparticles and blood components. Detailed evaluation of hemolytic potential, membrane integrity, oxidative stress induction, immunogenicity and protein corona analysis is required to ensure that the interaction between nanoparticles and erythrocytes does not trigger adverse hematological responses [42,46]. This protein corona influences the nanoparticles’ biological identity, circulation time, cellular uptake, and overall therapeutic or toxic effects. The layer of bound proteins can affect how immune cells recognize it, how efficiently it reaches target tissues, and how it interacts with cells at the molecular level. Understanding and controlling the formation of the plasma protein corona is therefore critical for designing nanoparticles with predictable behavior and improved safety in biomedical applications [47]. Only through meticulous delineation of these parameters can the full therapeutic potential of erythrocyte-based nanocarriers be safely and effectively realized.

### 3.4. Interaction with Erythrocyte Membranes and Laboratory Techniques to Identify the Modifications

The study of interaction between nanoparticles and red cell membranes is of utmost importance for the design of an appropriate drug delivery system using erythrocyte membrane as camouflage [48]. In detail, the erythrocyte membrane is primarily composed of a phospholipid bilayer interspersed with integral and peripheral membrane proteins, underpinned by an underlying cytoskeletal network. The lipid bilayer consists of asymmetrically distributed phospholipids, including phosphatidylcholine, sphingomyelin, phosphatidylethanolamine, phosphatidylserine, and phosphatidylinositol, which contributes to membrane fluidity and functionality [49,50,51]. Cholesterol is also embedded within the bilayer, modulating membrane stability and permeability. Membrane proteins such as band 3, glycophorins, and various ion transporters facilitate gas exchange, ionic balance, and cell recognition. The cytoskeletal framework, primarily composed of spectrin, actin, ankyrin, and protein 4.1, provides mechanical resilience and maintains the characteristic biconcave shape of erythrocytes, enabling their deformability and efficient passage through microvasculature [52,53].

When nanoparticles are embedded within erythrocyte membranes, the integration process involves specific physicochemical interactions between the nanoparticle surface and the lipid bilayer components of the membrane. These interactions can occur via hydrophobic forces, electrostatic interactions, van der Waals forces, or covalent modifications, depending on the surface functionalization of the nanoparticles. The incorporation is typically achieved through methods such as co-extrusion, sonication, or hypotonic dialysis, which temporarily disrupt the membrane to facilitate nanoparticle entry, followed by membrane resealing to restore integrity [54].

To evaluate the interaction between nanoparticles and erythrocytes, a range of advanced imaging and analytical techniques are employed. The relative spatial localization of nanoparticles in relation to erythrocyte membranes can be effectively visualized using laser scanning microscopy (LSM) and transmission electron microscopy (TEM) [55]. These techniques provide high-resolution images that reveal the precise distribution and membrane association of nanoparticles, allowing for the assessment of their binding patterns and localization. Additional techniques commonly used in this area of research include atomic force microscopy (AFM), which provides topographical information at the nanoscale, Raman spectroscopy for molecular fingerprinting, and confocal fluorescence microscopy, which enables real-time visualization of fluorescently labeled nanoparticles within or on erythrocytes. To simulate and understand the molecular forces governing nanoparticle erythrocyte interactions, computational and experimental models highlight the importance of electrostatic interactions, hydrophobic forces, and unique surface characteristics of nanoparticles. These forces collectively dictate the adhesion, penetration, or internalization of nanoparticles into the erythrocyte membrane [56].

In detail, the location of nanoparticles into erythrocytes can be visualized by laser scanning electron microscopy (LSM) and transmission electron microscopy (TEM) that mainly focuses on location information of nanoparticles compared to erythrocytes. Other methods widely used in research are atomic force microscopy (AFM), Raman spectroscopy, and confocal fluorescence microscopy. As regards the simulation of the molecular forces developing, it seems that electrostatic action, hydrophobic force, Coulomb attraction as well as the unique characteristics of nanoparticles play important roles in their interaction with erythrocyte membranes. Also, NMR (Nuclear Magnetic Resonance) technology, the self-diffusion coefficients of fullerene molecules (a nanomaterial that has wide use in biomedical research and especially in drug delivery), is linked to the lateral diffusion coefficients of erythrocyte membrane lipids via hydrogen bonds, which means that derivatives of fullerene molecules can be absorbed by erythrocytes and either bind to their membrane or enter interior [57]. The interaction of nanoparticles with membrane scaffold proteins can affect erythrocyte morphology, causing effects and changes in their membrane integrity, fluidity, deformability, and permeability [55]. The permeability and integrity of red blood cell membranes are typically assessed by measuring the activity of intracellular enzymes, such as lactate dehydrogenase (LDH), released into the extracellular space, as well as by quantifying potassium levels in the supernatant and evaluating hemolytic activity. Using the voltage clamp technique, a method employed to measure ionic currents across cell membranes, Djayanti and her colleagues investigated the effects of 3 μM fullerenol C_60_(OH)_24_ added to both sides of a lipid bilayer membrane (BLM). Their findings revealed that fullerenol C_60_(OH)_24_ aggregates in a conductive state, inducing ionic permeability and forming ionic pores, predominantly cation-selective, in the lipid bilayer. Moreover, the presence of cations such as calcium and magnesium ions at concentrations around 10^−3^ M was found to reduce the fullerenol C_60_(OH)_24_-induced permeability to potassium ions [58]. Also, acid functionalized single walled carbon nanotubes (AFSWCNTs) can interact directly with the erythrocyte membrane or enter the cytoplasm, thus leading to significant membrane damage and externalization of phosphatidylserine. This process occurs rapidly and varies depending on the dose administered and the duration of administration [59]. From the above, it is evident that red blood cells (RBCs) can serve as a fundamental platform for the development of effective drug delivery systems.

However, a detailed review of the interactions between RBCs and various nanoparticles loaded with active pharmaceutical ingredients reveals that, in many cases, the survival of the carrier cells is compromised. This is often due to alterations in their basic morphology or changes in the expression of surface molecules and receptors [60]. Thus, despite the considerable advantages this approach offers, the associated risks cannot be overlooked. In response, researchers have explored alternative strategies, studying the potential of erythrocytes to contribute to the safe and effective transport and delivery of nanoparticles containing active substances [61]. Numerous experiments and primarily in vitro studies have been conducted to create nanoparticles enclosed within isolated erythrocyte membranes, aiming to preserve the functional integrity of the RBC-derived carriers while maximizing therapeutic efficacy [62,63]. In conclusion, taking into consideration all the previous research articles, some disadvantages of the specific nanoparticles are the process of isolating RBC membranes and coating nanoparticles requiring meticulous techniques, which can be labor-intensive and time-consuming. Also, translating laboratory-scale methods to large-scale production remains challenging due to the complexity and cost of the procedures. Maintaining the structural integrity of the RBC membrane during preparation is crucial as any damage can compromise the functionality of the nanoparticles. Regarding the advantages, the laboratory methodologies used to prepare RBC-derived nanoparticles are highly versatile because they can be adapted to carry a wide variety of NPs. This versatility is largely due to the modular nature of the preparation methods, which allows the compound to be encapsulated in the nanoparticle core, adsorbed onto the surface, or embedded within the RBC membrane.

### 3.5. Clinical Application in Anticancer Therapy

**Figure 2:**
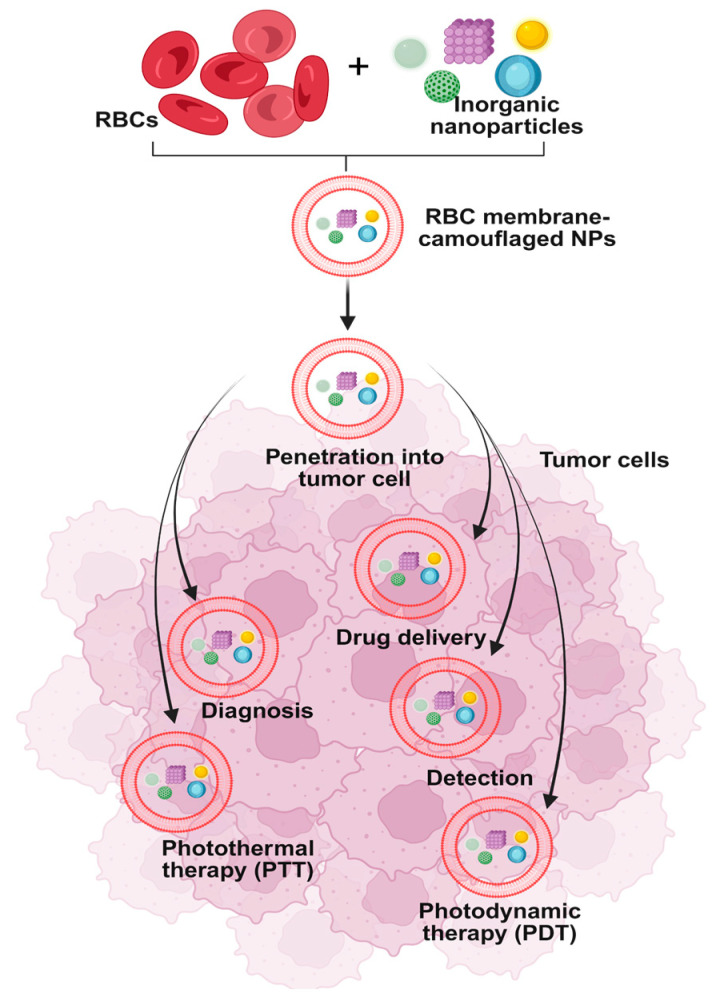
Applications of inorganic nanoparticles (INPs) in the diagnosis and treatment of cancer through photodynamic therapy, photothermal therapy, and drug delivery. Created in BioRender. Fortis, S. (2025) https://BioRender.com/0645avy.

Erythrocyte membrane-camouflaged anticancer nanoparticle delivery systems have demonstrated versatility across multiple therapeutic modalities beyond traditional chemotherapy for the treatment of neoplasms and solid tumors [64]. Among the most promising approaches are photodynamic therapy (PDT), photothermal therapy (PTT), and immunotherapy [65] (Figure 2). Specifically in PDT, the intrinsic oxygen-carrying capacity of erythrocytes can be strategically exploited to enhance therapeutic efficacy. The erythrocyte membrane encapsulating the nanoparticles not only preserves but also promotes the adsorption and transport of molecular oxygen at the nanoparticle surface, thereby facilitating the generation of reactive oxygen species upon light activation, an essential mechanism underlying photodynamic cytotoxicity. With regard to PDT, it is possible to effectively utilize the exceptional ability of erythrocytes to bind and transport large amounts of oxygen, as the erythrocyte membrane in which the nanoparticles are enclosed allows and promotes the penetration and binding of molecular oxygen on its surface [66]. In a study based on PDT, mitochondrial sites were targeted at the intracellular level, combining the properties of the membrane (for the purpose of distinct and selective recognition of cancer cells) and mitochondria, in order to achieve appropriate targeting of cancer cells [67]. In another study, another complex of manganese dioxide nanoparticles was constructed, which were covered by erythrocyte membranes, which functioned as an oxygen precursor, towards the final formation of oxygen and relief from the induced tumor hypoxia. Thus, it was observed that the coating of the erythrocyte membrane allowed the increased loading capacity of the drug Dox, while its release to the target cancer cells was facilitated and accelerated through the rupture of the membrane, which was caused by the oxygen produced [68]. Another approach based on the mimicry technique involves the combination of PTT and chemotherapy treatments, utilizing a good photothermal agent, Prussian blue dye. Multifunctional nanoparticles coated with the erythrocyte membrane have shown their ability to play an essential role in inhibiting tumor growth [69]. Recently, artificial cells have been developed by encapsulating a nanogel consisting of methylene blue (MB) and cisplatin inside the erythrocyte membrane, which are inspired by the natural clearance mechanisms of cytotoxic T lymphocytes (CTLs) [70]. These artificial cells are regulated in such a way as to accurately mimic the function of CTLs. Specifically, when these come into contact with cancer cells and are irradiated, they create slits or pores on the surface of the target cells, which are caused by the photoheating of the MB. This sequence of events leads to the release of the therapeutic MB and cisplatin, having a more beneficial effect on the fight against malignancies by combining the benefits of photodynamic therapy, photothermal therapy, and chemotherapy, drastically limiting the toxicity caused by chemotherapy itself [71]. Building on these strategies, the interaction of nanoparticles with erythrocyte membranes is a critical factor for their efficacy and safety. Laboratory techniques such as electron microscopy, dynamic light scattering, and surface protein assays allow precise characterization of membrane coating and any modifications, ensuring the nanoparticles retain the functional properties of the erythrocytes. This careful engineering enhances hemocompatibility, minimizing adverse interactions with blood cells and plasma proteins. Loading erythrocytes with nanoparticles also provides significant advantages, including prolonged circulation time, immune evasion, and the ability to deliver multiple therapeutic agents simultaneously. By integrating principles of nanotechnology, researchers can design multifunctional nanoparticles that exploit the natural properties of erythrocytes, achieving targeted drug delivery, improved oxygen transport for photodynamic therapies, and controlled release of chemotherapeutics. Collectively, these approaches demonstrate how biomimetic erythrocyte-based nanoparticles can combine safety, versatility, and efficacy, establishing a robust platform for advanced anticancer therapies.

## 4. Discussion

Nanoparticle-based drug delivery strategies have demonstrated exceptional efficacy in the targeted and site-specific release of therapeutic agents. Table 1 presents the key findings of our literature review identifying the key findings of the selected articles.

This can be attributed to their distinctive ability to provide insights about NPs physical, chemical, and biological properties, which enable precise delivery, enhanced bioavailability, and reduced systemic toxicity. In oncology, nanodevices are extensively employed to transport chemotherapeutic agents directly to tumor cells and microenvironments, thereby improving therapeutic outcomes and minimizing off-target effects [72] (Figure 2). A significant proportion of these investigational nanomedicines are in early clinical development, with the majority in phase I and phase II trials. The principal areas of focus include the treatment of cancer and infectious diseases, which together account for a substantial percentage of ongoing research and development efforts [73]. Among the various nanomedicine platforms, liposomal and lipid-based nanoparticles are the most prevalent, representing the largest share of both approved and investigational products [74]. Other widely studied nanocarriers include polymer-drug and polymer-protein conjugates, antibody-drug conjugates, polymeric nanoparticles, and viral vectors. Additionally, emerging delivery systems such as cell-derived vesicles, inorganic nanoparticles, emulsions, protein-based nanoparticles, micelles, nanocrystals, and dendrimers are actively being investigated for clinical translation [75]. In our review, we specifically highlighted the use of erythrocytes as natural, biocompatible vehicles for nanoparticle delivery, presenting current research advances and mechanistic insights into their application in nanomedicine. By leveraging the unique biological properties of red blood cells such as long circulation half-life, immune evasion, and membrane flexibility, we explored their potential as versatile and safe platforms for targeted drug delivery. At present, organic nanomaterials such as liposomes and polymeric micelles constitute the majority of FDA-approved nanomedicines and those under active clinical investigation, particularly for the treatment of hematological malignancies [76]. These organic carriers are favored for their biocompatibility, biodegradability, and ability to encapsulate a wide range of therapeutic agents. The delivery of pharmaceutical substances to specific targets within the human body such as cells, tissues, and organs is a critical factor influencing patient prognosis [77]. While conventional drug delivery methods have greatly contributed to the treatment of various diseases, they often fall short in terms of effective absorption and targeted release, frequently leading to unwanted side effects [77]. The use of nanoparticles as carriers of active substances has revolutionized therapeutic strategies across a wide range of pathologies. Nanoparticles can be engineered to carry specific receptors on their surface, enabling them to bind selectively to target cells based on disease-specific mechanisms [76]. However, a major limitation of this approach lies in the immune response they trigger; nanoparticles are often recognized as foreign bodies, provoking immune attacks that can cause systemic complications and, in some cases, render the therapy unsuitable [78]. Delivery systems leveraging the unique properties of red blood cells (RBCs) offer promising solutions to these challenges. Based on the presented data, RBC-based systems show significant advantages over traditional delivery methods. A relatively recent development in this field is the creation of biomimetic nanoparticles encapsulated within cell membranes that have been isolated from their intracellular components. These biomimetic systems mimic the physiological functions of native cells. Their principal benefits include immune system recognition as ‘self,’ which minimizes toxicity, and a controlled, highly specialized release of the pharmaceutical agent, attributed to their prolonged circulation in the bloodstream.

**Table 1:** Summary of research articles and review summarizing the results and the final applications of different drug carrier types.

Nanoparticle Composition (Drug Carrier Type)	Production Method	Results	Final Application	References
Polystyrene nanoparticles	Physical adsorption onto erythrocyte surface	Prolonged circulation time depending on NP size; RBC morphology largely maintained	Long circulating drug delivery system	[31]
Polymer–protein conjugate carriers	PEG-coated liposomes	Improved solubility; reduced solvent-associated toxicity; enhanced tumor penetration	Clinically approved for metastatic breast cancer, pancreatic cancer	[32]
Biodegradable polymeric nanoparticles (e.g., PLGA core) coated with natural erythrocyte membrane	Top-down biomimetic coating: hypotonic lysis of RBCs, continously → membrane vesicle preparation and then→ fusion (e.g., extrusion) onto polymeric nanoparticle	Significantly prolonged circulation half-life in mice; high retention at 72 h post-injection compared with PEGylated control particles	Long-circulating drug delivery platform that evades immune clearance and improves systemic retention	[60]
Polymer–drug conjugates	Covalent conjugation: Attaching drugs or proteins to polymers through stable covalent bonds.	Enhanced solubility and stability: conjugation improves the solubility and stability of poorly water-soluble drugs and proteins.	Cancer therapy: polymer–drug conjugates are utilized for targeted chemotherapy, reducing systemic toxicity.	[75]
Cell membrane coated nanoparticles	Extrusion	Immune evasion; prolonged circulation	Targeted cancer therapy	[70]
Erythrocyte-inspired functional materials (e.g., RBC membrane-coated nanoparticles, erythrocyte-mimicking carriers)	Biomimetic engineering approaches: coating synthetic nanoparticles with RBC membranes, constructing erythrocyte-mimetic systems	Prolonged circulation time, immune evasion, improved biocompatibility, targeted drug delivery	Drug delivery, biosensing, detoxification, and other biomedical applications	[68]

## 5. Conclusions

This paper has reviewed the benefits of using erythrocyte membranes as a template for constructing nanoparticle–drug complexes, highlighting their advantages compared to other delivery techniques. One of the most actively researched applications of this approach is in oncology, where nanoparticles encapsulated in erythrocyte membranes may offer a more favorable prognosis compared to conventional chemotherapy. Further optimization of these methods could pave the way for effective treatments or even radical interventions in the fight against malignant tumors. In conclusion, biomimetic nanoparticles based on red blood cell properties represent a “smart” unique and innovative strategy for drug delivery. With continued refinement and in-depth study of their structure, assembly, and safety profiles, they hold great promise for treating a wide range of diseases more safely and effectively.

## Data Availability

Data is contained within the article.
